# CRABP-II enhances pancreatic cancer cell migration and invasion by stabilizing interleukin 8 expression

**DOI:** 10.18632/oncotarget.14194

**Published:** 2016-12-26

**Authors:** Shuiliang Yu, Neetha Parameswaran, Ming Li, Yiwei Wang, Mark W. Jackson, Huiping Liu, Wei Xin, Lan Zhou

**Affiliations:** ^1^ Department of Pathology, Case Western Reserve University, Cleveland, Ohio, USA; ^2^ Biostatistics and Bioinformatics Core Facility, Case Comprehensive Cancer Center, School of Medicine, Case Western Reserve University, Cleveland, Ohio, USA; ^3^ Department of Pathology, University Hospitals Case Medical Center, Cleveland, Ohio, USA

**Keywords:** pancreatic cancer, CRABP-II, IL-8, MMP-2/MMP-14, metastasis

## Abstract

Our previous study shows that cellular retinoic acid binding protein II (CRABP-II) is overexpressed in pancreatic ductal adenocarcinoma (PDAC) and pre-cancerous lesions, but not detected in normal pancreatic tissues. In this study, we show that deletion of CRABP-II in PDAC cells by CRISPR/Cas9 does not affect cancer cell proliferation, but decreases cell migration and invasion. Gene expression microarray analysis reveals that IL-8 is one of the top genes whose expression is down-regulated upon CRABP-II deletion, while expression of MMP-2 and MMP-14, two targets of IL-8 are also significantly down-regulated. Moreover, we found that CRABP-II is able to form a complex with HuR, which binds to the 3′UTR of IL-8 messenger RNA (mRNA) and enhances IL-8 mRNA stability. Ectopic expression of flag-CRABP-II in CRABP-II knockout cells is able to rescue the expression of IL-8, MMP-2/MMP-14 and recovers cell migration. Using the orthotopic xenograft model, we further demonstrate that CRABP-II deletion impairs tumor metastasis to nearby lymph nodes. Taken together, our results reveal a novel pathway linking CRABP-II expression to enhanced PDAC metastasis, and hence we propose CRABP-II may serve as a new PDAC therapeutic target.

## INTRODUCTION

Pancreatic cancer is one of the major causes of cancer death worldwide [1] and in the US [2]. Pancreatic ductal adenocarcinoma (PDAC) is the most common type of pancreatic cancer, with an overall 5-year survival rate at around 5% [3]. The ability of PDAC to metastasize in early stages is regarded as the primary reason for its notoriously dismal clinical outcome. Thus, identifying new therapeutic targets critically implicated in PDAC progress and metastasis has become a key task for pancreatic cancer researchers.

Cellular retinoic acid binding protein II (CRABP-II) is a soluble small protein residing in the cytosol. It belongs to the family of intracellular lipid-binding proteins and binds all-trans retinoic acid (RA), a vitamin A metabolite with powerful transcriptional activities at a sub-nM affinity [4, 5]. Upon binding to RA, CRABP-II translocates to the nucleus and delivers the ligand to retinoic acid receptor (RAR), thus facilitating its transcriptional activation and affecting cell proliferation, differentiation and apoptosis [6, 7]. Recently, CRABP-II was found to be involved in the regulation of mRNA stability by its interaction with HuR, a well-known RNA binding protein in mammals [8]. However, whether this property of CRABP-II is related to its function in cancer particularly in PDAC has not been explored.

CRABP-II expression has been reported in a wide variety of human cancers including breast, ovarian, prostate, bladder tumors, neuroblastoma, glioma, head and neck squama, acute promyelocytic leukemia, and renal cell carcinoma. In fact, CRABP-II expression is either up-regulated in some cancer types or down-regulated in other forms [9–20]. In MMTV-Neu breast cancer mouse model, CRABP-II is defined as a tumor suppressor because of its ability to enhance RAR activation to promote cell cycle rest, differentiation and apoptosis [21, 22]. However, in ER-negative breast tumors, CRABP-II overexpression is associated with a reduced overall survival [9]. The same correlation is also found in Wilms tumors [12, 23, 24]. Further, CRABP-II expression is higher in melphalan-resistant MCF-7 breast cancer cell line than in the susceptible MCF-7 cells [25], suggesting a role of CRABP-II in drug resistance. Notably, ectopic expression of CRABP-II in neuroblastoma cells increases cell motility [11], while CRABP-II down-regulation in head and neck squamous cell carcinoma results in a decrease of cell invasion [26], indicating that CRABP-II might be involved in cell mobility regulation. The diverse function of CRABP-II in various cancers may be explained by the tissue-specific or cell-context-dependent differential roles of CRABP-II. However, the mechanism by which CRABP-II facilitates tumor motility and invasion remains unknown.

Our recent study showed that all the primary PDAC tumors and a portion of pre-cancerous lesions over-express CRABP-II, while neither normal pancreatic parenchyma nor ductal epithelium or chronic pancreatitis express CRABP-II [27]. In current study, we report that CRABP-II enhances cancer cell migration and invasion through regulating IL-8/MMP-2/MMP-14 pathway.

## RESULTS

### Silencing or knockout of CRABP-II reduced PDAC cell migration and invasion without affecting cell proliferation

**Figure 1:**
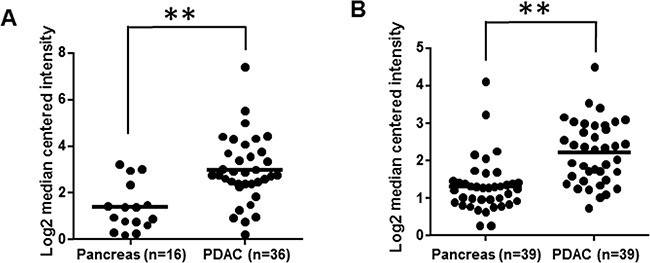
Overexpression of CRABP-II in pancreatic cancer **A.** CRABP-II expression in Pei Pancreas microarray database from Oncomine; ** p = 1.86E-5 by student t-test. **B.** CRABP-II expression in Badea Pancreas microarray database from Oncomine; ** p = 1.33E-6 by student t-test.

Our recent report of PDAC-specific overexpression of CRABP-II agrees with the RNA array results from Oncomine databases (Figure 1A and 1B) [27]. Notably, higher level of CRABP-II expression was detected in metastatic lymph nodes [27], suggesting that CRABP-II may contribute to PDAC metastasis. Studies of PDAC cell lines (BxPC-3, Capan-1, Panc-1 and Panc 10.05) revealed that CRABP-II is highly expressed in all these cells (Supplementary Figure 1). Considering its progressively increased expression in PanIN1 through PanIN3, and its high level in metastatic lymph nodes, we speculate that CRABP-II may function as a tumor promoter for PDAC.

**Figure 2:**
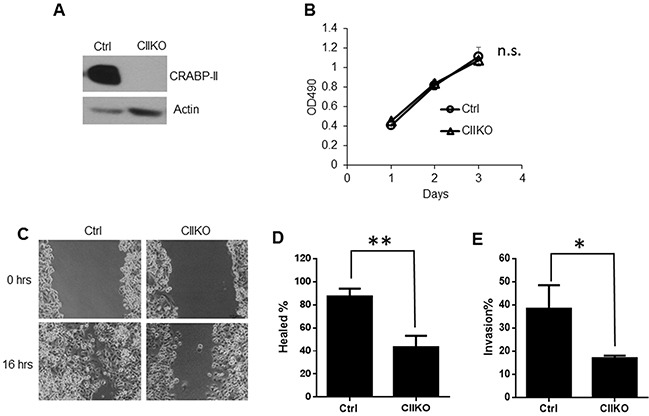
Reduction of cell migration and invasion by CRABP-II deletion **A.** Knockout of CRABP-II in Panc-1 cells shown by western blots. **B.** MTT assays showing no change in cell proliferation after deletion of CRABP-II. Data shown represent the means ± SD of triplicate wells from three independent experiments. n.s. means no significant difference by one-way AVONA test. **C.** and **D.** Wound healing assays showing the decrease of cell migration in CRABP-II KO cells. Data shown represent the means ± SD of three independent experiments. ** p< 0.01 by student t-test. **E.** Matrigel invasion assays showing cell invasiveness reduction in CRABP-II KO cells. Data shown is the means ± SD of three independent experiments. * p<0.05 by student t-test.

To investigate this hypothesis, we first down-regulated CRABP-II expression by shRNA in Panc-1 cells, which have relatively higher CRABP-II expression level (Supplementary Figure 1). Immunoblots and qRT-PCR showed that CRABP-II expression was down regulated by 80-90% in CRABP-II knockdown (KD) cells (Supplementary Figure 2A and 2B). Down-regulation of CRABP-II, however, did not affect cell proliferation (Supplementary Figure 2C), but significantly decreased cell mobility in both wound healing and Matrigel invasion assays (Supplementary Figure 2D and 2E). These results indicate that CRABP-II is mainly involved in the regulation of PDAC metastatic behaviors. Because CRABP-II is not expressed in normal pancreas, we then evaluated the effects of CRABP-II knockout (KO) using CRISPR/Cas9 (Figure 2A). Consistently, CRABP-II KO Panc-1 cells had a comparable proliferation (Figure 2B) but lower migration (Figure 2C and 2D) and invasion (Figure 2E), when compared to CRISPR negative control cells.

**Figure 3:**
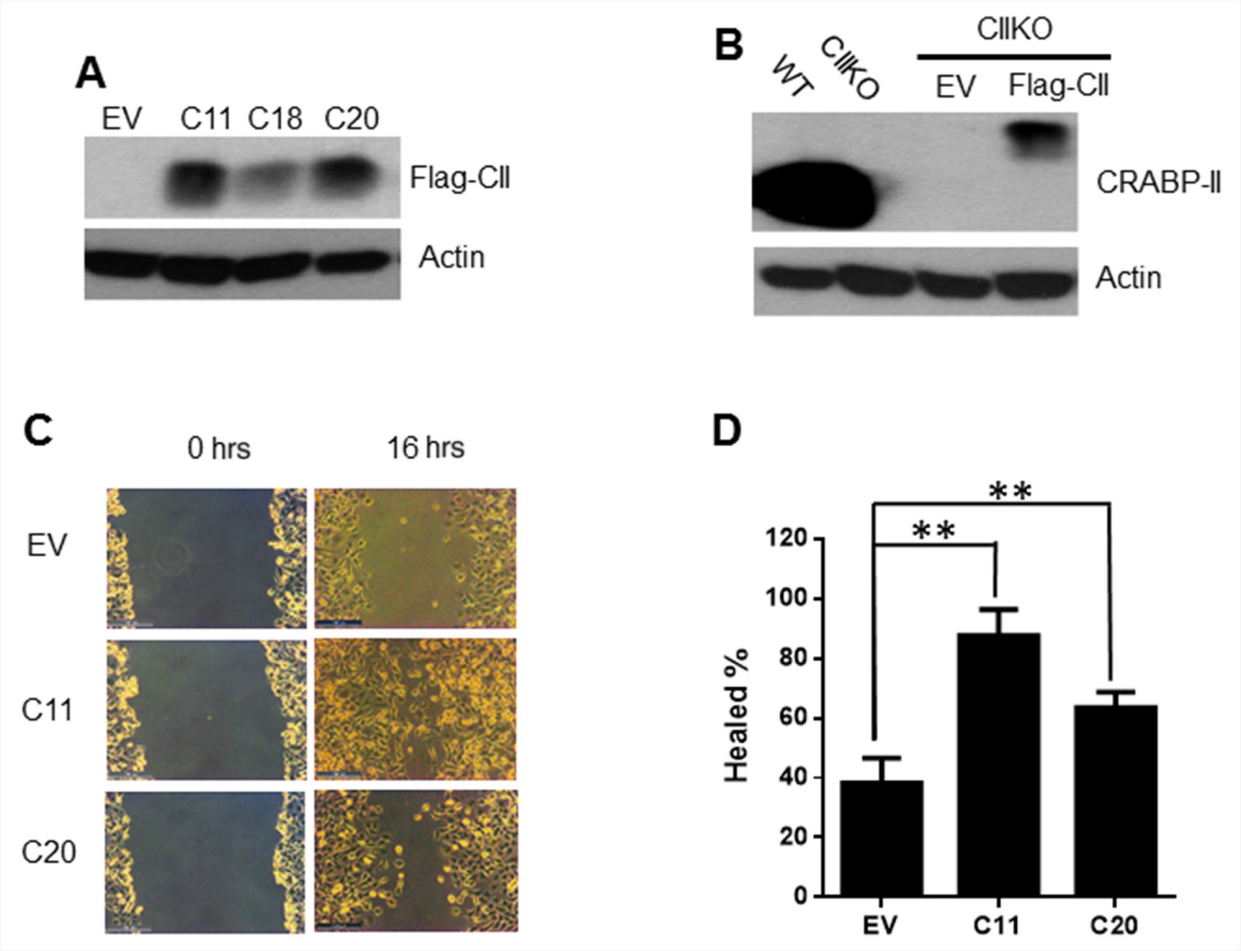
Rescuing cell migration by re-expressing CRABP-II in knockout cells **A.** Expression of flagged CRABP-II in KO cells shown by western blot with anti-flag antibody. EV, empty vector transfected; C11, C18 and C20 are three independent clones established. **B.** Comparison between endogenous CRABP-II and ectopic expression of flagged CRABP-II by western blot with anti-CRABP-II antibody. **C.** and **D.** Wound healing assays showing the increase of cell migration in flag-CRABP-II expressing cells. Data in (D) represent the means ± SD of three independent experiments. ** p< 0.01 vs. empty vector by student t-test.

To confirm that the phenotype observed in CRABP-II KO cells is indeed caused by CRABP-II deficiency, we transfected a construct of 3xflagged CRABP-II with site-directed mutation at the PAM sequence of Cas9 nuclease (TGG to TCG), back into CRABP-II KO cells (Figure 3A). A pool of 3 clones was used in the rescue experiment. Although the expression of flagged CRABP-II in rescued cells is just about 20% of the endogenous CRABP-II in unmodified PDAC cells (Figure 3B), it was able to increase the migration of CRABP-II KO cells (Figure 3C and 3D), demonstrating that CRABP-II indeed facilitates PDAC cell motility.

### Deletion of CRABP-II decreased the expression of interleukin 8 (IL-8) in Panc-1 cells

**Figure 4:**
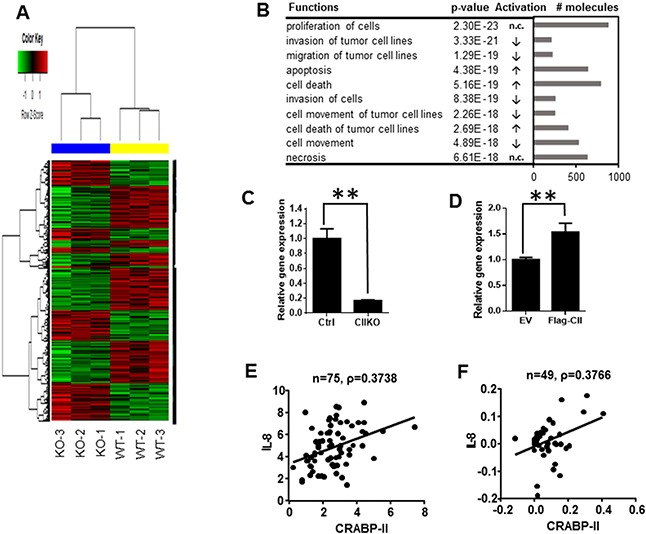
Down-regulation of IL-8 in CRABP-II knockout cells **A.** Heat map of gene expression microarray. 1895 down-regulated genes and 1478 up-regulated genes upon CRABP-II deletion in Panc-1 cells were shown. **B.** Molecular and cellular function analysis by IPA software (Qiagen). The top 10 functions were listed. The arrows in activation column indicate functions in CIIKO cells compared to WT cells, “increase (↑)”, “decrease (↓)” or “no change (n.c.)”. **C.** qRT-PCR showing decrease of IL-8 expression in CRABP-II KO cells. Data were normalized to actin mRNA and represent the means ± SD of three biological replicates. ** p<0.01 by student t-test. **D.** qRT-PCR showing increase of IL-8 expression in flagged CRABP-II expressing cells. Data were normalized to actin mRNA and represent the means ± SD of three biological replicates. ** p<0.01 by student t-test. **E and F.** Correlation between IL-8 and CRABP-II expression in human pancreatic cancer specimens by Pearson’s product-moment correlation coefficient analysis (PPMCC). Data shown in (E) are combination of Pei Pancreas and Badea Pancreas from Oncomine (n=75) and Pearson correlation coefficient number ρ= 0.3738; Data shown in (F) are from TCGA Pancreas in Oncomine (n=49) and Pearson correlation coefficient number ρ= 0.3766.

Next, in order to understand the molecular mechanism by which CRABP-II increases PDAC invasion and migration, we performed gene expression microarray analysis. Compared to CRISPR negative control cells, 1895 genes were down-regulated while 1478 genes were up-regulated (Figure 4A). Molecular and cellular function analysis revealed that both cell invasion and migration functions were decreased in CRABP-II KO cells (Figure 4B), while the functional status of cell proliferation was not affected by CRABP-II deletion. These findings are consistent with the cell culture observations. The top 20 genes that are down-regulated in CRABP-II KO cells are listed in Table 1. Although LPHN2 (Latrophilin 2) is the mostly down-regulated gene, its function has not been identified. IL-8 (interleukin 8), the second mostly down-regulated gene in the list, is well-known in cancer progression and its expression was decreased by 6 folds. Real time PCR confirmed that IL-8 was drastically decreased in CRABP-II KO cells (Figure 4C). Furthermore, re-expression of flagged CRABP-II in KO cells recovered suppressed IL-8 expression (Figure 4D), indicating that IL-8 is a downstream target of CRABP-II.

**Table 1:** Top 20 genes down-regulated by CRABP-II knockout in Panc-1 cells

ProbID	TargetID	P.Val	FC	Difinition
ILMN_1697548	LPHN2	5.87E-11	−8.35	Latrophilin 2
ILMN_2184373	IL8	5.81E-10	−6.04	Interleukin 8
ILMN_1752520	SLFN11	3.51E-09	−3.82	Schlafen family member 11
ILMN_1759330	KIF1A	7.14E-10	−3.79	Kinesin family member 1A
ILMN_1725193	IGFBP2	1.26E-09	−3.78	Insulin-like growth factor binding protein 2, 36kDa
ILMN_1787265	ZNF503	9.83E-10	−3.67	Zinc finger protein 503
ILMN_1733998	DHRS9	1.26E-09	−3.59	Dehydrogenase/reductase (SDR family) member 9, transcript variant 1
ILMN_1669617	GRB10	3.41E-09	−3.47	Growth factor receptor-bound protein 10, transcript variant 1
ILMN_1663575	MGC87042	2.86E-06	−3.44	Similar to Six transmembrane epithelial antigen of prostate
ILMN_1669046	FOXQ1	8.58E-10	−3.44	Forkhead box Q1 (FOXQ1)
ILMN_1691790	DACT2	9.38E-09	−3.4	Dapper, antagonist of beta-catenin, homolog 2 (Xenopus laevis)
ILMN_2352921	BPGM	3.83E-09	−3.35	2,3-bisphosphoglycerate mutase, transcript variant 2
ILMN_2202915	FAR2	1.49E-08	−3.3	Fatty acyl CoA reductase 2 (FAR2)
ILMN_2387385	IGFBP1	2.34E-08	−3.24	Insulin-like growth factor binding protein 1
ILMN_2060413	CD24	1.72E-09	−3.17	CD24 molecule
ILMN_1747650	BMP6	1.16E-08	−3.06	Bone morphogenetic protein 6
ILMN_1814221	NPTX1	2.78E-08	−3.03	Neuronal pentraxin I
ILMN_1810725	FAM129A	1.59E-07	−3.01	Family with sequence similarity 129, member A, transcript variant 2
ILMN_2121408	HBEGF	1.79E-08	−3	Heparin-binding EGF-like growth factor
ILMN_2181892	BEX2	2.12E-06	−2.94	Brain expressed X-linked 2

The up-regulation of IL-8 has been demonstrated in various human cancers, including breast, colon, and lung cancer [28, 29], as well as pancreatic cancer [30–32]. In order to test if IL-8 is a major mediator of CRABP-II on cell invasion and migration, we examined IL-8 expression in pancreatic cancer specimens from Oncomine databases. Compared with normal tissue, pancreatic tumors express higher level of IL-8 in both databases we examined (Supplementary Figure 3). Further, there is a moderate correlation between CRABP-II and IL-8 expression in pancreatic cancer in both database (Figure 4E and 4F), suggesting that CRABP-II may regulate IL-8 expression in pancreatic cancer.

### CRABP-II up-regulates IL-8 expression by stabilizing IL-8 mRNA through cooperating with HuR

**Figure 5:**
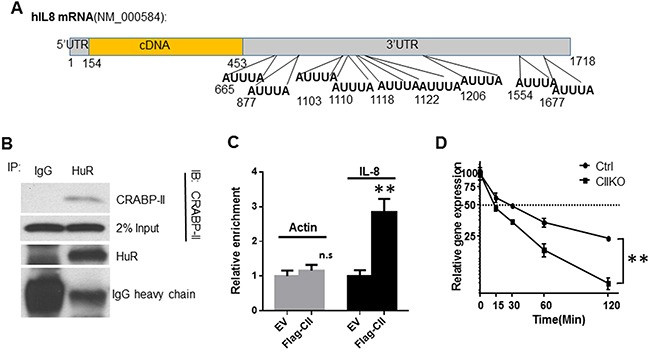
Stabilization of IL-8 mRNA by CRABP-II/HuR complex **A.** Schematic diagram of human IL-8 mRNA sequence. 9 putative AU rich elements (ARE) are shown in the 3′UTR region. **B.** Interaction between CRABP-II and HuR in Panc-1 cells. The cell lysates were pre-treated with 100 μg/ml RNase for 30 min. Immunoprecipitation was performed by using anti-HuR mAb (3A2) and mouse IgG was used as negative control. Anti-CRABP-II antibody was used for immunoblot. **C.** RNA immunoprecipitation (RIP) showing the enrichment of CRABP-II on IL-8 mRNA. Protein-RNA complexes were precipitated by anti-flag beads. RNA was isolated from beads and the binding of IL-8 mRNA was assessed by qRT-PCR. Actin mRNA was used as control. RNA isolated from 10% of input whole cell lysis was used as input control. Data represent the means ± SD of three independent experiments. **p<0.01 by student t-test. **D.** Half-life of IL-8 mRNA in CRABP-II knockout and control cells. Cells were pre-cultured in charcoal stripped media for 48 hrs followed by treatment with actinomycin D. RNA was isolated at denoted time points and the mRNA of IL-8 was assessed by qRT-PCR. Data represent the means ± SD of triplicate wells from three independent experiments. **, p<0.01 by one-way ANOVA test.

We then examined whether CRABP-II regulates IL-8 by interaction with HuR. It has been shown that HuR binds to and stabilizes IL-8 mRNA in both tumor cells [33] and normal epithelial [34]. Analysis of the 3′UTR region of IL-8 mRNA revealed 9 potential HuR binding motifs (ARE, AU rich element) (Figure 5A). Immunoprecipitation showed that CRABP-II forms a complex with HuR in Panc-1 cells (Figure 5B). RNase (100μg/ml) treatment did not affect the co-immunoprecipitation of CRABP-II with HuR, suggesting a direct protein-protein interaction. We then performed RNA immunoprecipitation (RIP) to investigate if CRABP-II/HuR complex binds to IL-8 mRNA in PDAC cells. CRABP-II KO cells expressing flagged CRABP-II (Flag-CII) were lysed, and the RNA bound to Flag-CII (pulled down by anti-flag beads) was isolated and assessed by qRT-PCR. Compared with empty vector transfected cells, Flag-CII expressing cells have 3-fold more of IL-8 mRNA recovered (Figure 5C). In comparison, although β-actin is a known target of HuR [35], there is no difference in recovered β-actin mRNA from Flag-CII expressing cells and control cells, suggesting that flag-CRABP-II specifically binds to IL-8 mRNA. To further examine the effect of this binding, cells were treated with actinomycin D and the half-life of IL-8 mRNA was assessed by qRT-PCR. We found that IL-8 mRNA in Panc-1 cells has a half-life of ∼30 min, whereas deletion of CRABP-II shortened its half-life to 14 min (Figure 5D). After 2 hours of actinomycin D treatment, IL-8 mRNA remained 33% of its original level in control cells but only 8% in CRABP-II KO cells. These results suggest that CRABP-II stabilizes IL-8 mRNA by interacting with HuR.

### Deletion of CRABP-II reduced MMP-2 and MMP-14 expression in Panc-1 cells

**Figure 6:**
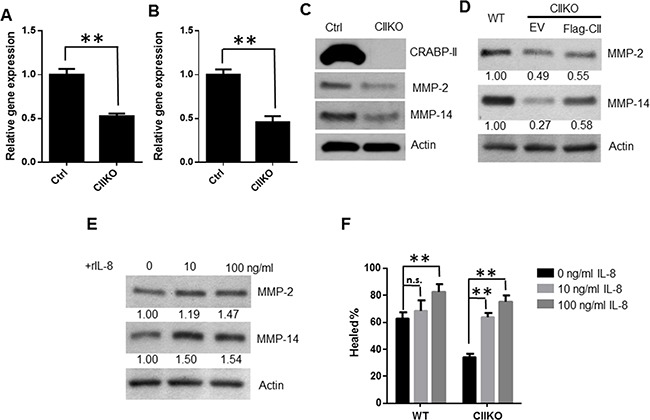
Reduction of MMP-2 and MMP-14 expression in CRABP-II knockout cells **A and B.** qRT-PCR showing the reduction of MMP-2 (A) and MMP-14 (B) expression in CRABP-II KO cells. Data represent the means ± SD of triplicate wells from three biological replicates. ** p<0.01 by student t-test. **C.** Western blots showing the reduction of MMP-2 and MMP-14 expression in CIIKO cells. **D.** Western blots showing the rescued MMP-2 and MMP-14 expression by ectopic expression of flag-CRABP-II. Blot density was quantified by using ImageJ software and normalized to actin and denoted under the pictures. **E.** Induction of MMP-2 and MMP-14 expression by IL-8 in CRABP-II KO cells. Cells were cultured in media with 1% FBS and treated with recombinant IL-8 at denoted concentrations for 48 hrs. Cells were lysed and MMP-2, MMP-14 expression were detected by western blots. The blot density was quantified by using ImageJ software and denoted under the pictures. **F.** Wound healing assays showing increase of cell migration by IL-8 treatment. Cells were cultured in media with 1% FBS and treated with different concentrations of IL-8. Cell monolayers were wounded with a plastic tip after the addition of IL-8. New media with IL-8 was supplemented for further 16 hrs. Data represent the means ± SD of three biological replicates. **p<0.01 vs. untreated by student t-test.

Because two matrix metalloproteinases (MMPs), MMP-2 and MMP-9, are overexpressed in many types of cancers and play key roles in tumor metastasis [36, 37], and IL-8 is able to induce MMP-2 and MMP-9 production [28], we wanted to examine if CRABP-II enhances cell migration and invasion through IL-8 mediated MMP-2 and MMP-9 induction. Compared to control cells, we found MMP-2 mRNA expression as well as protein level were decreased by 50% in CRABP-II KO (Figure 6A, 6C) or CRABP-II KD cells (Supplementary Figure 4A, 4E). MMP-14 (MT1-MMP), which activates pro-protein MMP-2, is also decreased close to 60% and 30% in KO (Figure 6B, 6C) and KD cells (Supplementary Figure 4C and 4E), respectively. However, another regulator of MMP-2, the metalloproteinase inhibitor 2 (TIMP-2), which inhibits MMP-2 activity showed no significant change (Supplementary Figure 4D). In comparison, MMP-9 has low expression in Panc-1 cells (data not shown) and it was slightly down-regulated in CRABP-II KD cells (Supplementary Figure 4B).

To further confirm that MMP-2 and MMP-14 reductions are caused by CRABP-II depletion, rescue experiments were performed. Indeed, just as Flag-CII increased cell migration (Figure 3C and 3D), Flag-CII expression in CRABP-II KO cells partially restored MMP-2 and MMP-14 expression (Figure 6D). These results suggest that CRABP-II facilities PDAC cells migration and invasion by regulating MMP-2/MMP-14 activity.

### IL-8 treatment rescued MMP-2/MMP-14 expression and cell migration in CRABP-II deficient cells

To further examine if regulation of MMP-2/MMP-14 by CRABP-II is mediated through IL-8, we asked whether IL-8 treatment could rescue the downregulated MMP-2/MMP-14 in CRABP-II KO cells. CRABP-II KO cells were cultured in low FBS media and treated with various doses of recombinant IL-8 for 48 hrs, and cell lysis were analyzed by immunoblots. We found both MMP-2 and MMP-14 expression increased in IL-8 treated cells when compared to untreated cells (Figure 6E). IL-8 treatment enhanced both control and KO cell migration in a dose-dependent manner (Figure 6F). In CRABP-II KO cells, 10 ng/ml IL-8 treatment is sufficient to rescue cell migration to the level seen in WT cells. These results suggest that IL-8 treatment is able to rescue the phenotype caused by CRABP-II deletion and that IL-8 is a major mediator of CRABP-II and acts upstream of MMP-2/MMP-14 on PDAC cell motility.

We then examined the possibility if IL-8 and MMP-2/MMP-14 are regulated by CRABP-II-mediated RA effect since IL-8 production could be induced by RA treatment [38]. Despite that RA successfully upregulated RARβ expression, RA treatment neither increased IL-8 nor increased MMP-2/MMP-14 expression (Supplementary Figure 5). These findings suggest that both IL-8 expression and MMP-2/MMP-14 activity are RA independent in PDAC cells.

### Silencing CRABP-II decreased other PDAC cell migration by reducing IL-8/MMP-2/MMP-14 signaling

Considering the diversity of phenotype and genotype of various PDAC cell lines, we examined if CRABP-II also up-regulates IL-8/MMP-2/MMP-14 signaling in other PDAC cells. We knocked down CRABP-II in BxPC-3, a metastatic PDAC line expressing wild type KRAS, and in Panc10.05, a less invading cell line expressing KRAS mutant (Supplementary Figure 6A). In both cell lines, the expression of IL-8 was decreased upon CRABP-II silencing (Supplementary Figure 6B and 6C). Silencing CRABP-II also reduced both MMP-2 and MMP-14 levels in BxPC-3, and down-regulated MMP-2 expression in Panc10.05 as there is no detectable MMP-14 expression (Supplementary Figure 6A). BxPC-3 cells showed decreased cell migration after CRABP-II silencing (Supplementary Figure 6D and 6E). However, Panc10.05 could not be evaluated by the same assay as these cells display very slow migration (data not shown). These results confirmed our observations in Panc-1 cells that CRABP-II enhances PDAC cells migration through increasing IL-8/MMP-2/MMP-14 expression.

### Deletion of CRABP-II impairs tumor metastasis in pancreatic orthotopic models

Lastly, to examine if expression of CRABP-II augments cell invasion and migration *in vivo*, we performed pancreas orthotopic xenografts analysis. Successful implantations were obtained in 6 control cell-injected mice and in 8 CRABP-II KO Panc-1-injected mice. Tumor growth and metastasis were monitored by bioluminescence imaging (BLI) every two weeks after surgery. We found that although the average bioluminescence of CRABP-II KO tumors was comparable to that of WT tumors, fewer signals were seen in the nearby organs in mice receiving CRABP-II KO cells than in mice receiving control cells (data reviewed but not shown). At 11 weeks after surgery, we found more nearby metastatic foci in mice receiving control cells than in mice receiving CRABP-II KO cells (data reviewed but not shown). Lymph node metastases were observed in 3 mice of control group while none was found in CRABP-II KO injected mice (Supplementary Figure 7). These results agree with observations in cell models and demonstrate that deletion of CRABP-II impairs tumor metastasis *in vivo*.

## DISCUSSION

In this study, we investigated the role of CRABP-II in PDAC migration and invasion. Either knockdown CRABP-II by shRNA or knockout CRABP-II by CRISPR/Cas9 decreases PDAC cell migration and invasion, but has no effect on cell proliferation. Deletion of CRABP-II drastically reduces IL-8 expression. Reduction of IL-8 by CRABP-II deletion attenuates MMP-2 and MMP-14 expression, resulting in the impairment of cell motility. Mechanistically, CRABP-II regulates IL-8 expression by forming HuR/CRABP-II complex that binds to the 3′UTR of IL-8 mRNA, stabilizing IL-8 messenger and promoting IL-8 expression. Finally, CRABP-II deletion in pancreatic orthotopic xenografts decreases PDAC local invasion to lymph nodes, supporting the role of CRABP-II as a critical regulator of PDAC migration and invasion.

Distinct from the well-characterized function of CRABP-II to deliver RA from cytosol to nucleus to activate RA receptor-dependent transcription, results from this study reveals that one of the CRABP-II functions in PDAC cells implicates IL-8 mRNA stabilization through CRABP-II/HuR complex bound to the 3′UTR region of IL-8. IL-8 mRNA has been identified as a target of HuR in both tumor cells [33] and normal epithelial [34]. However, IL-8 mRNA contains 9 putative HuR binding motifs (AREs) in its 3′UTR (Figure 5A). Which ARE is the critical binding motif for CRABP-II/HuR to interact with IL-8 mRNA remains to be determined. In addition, it warrants further investigation if up-regulation of IL-8 by CRABP-II/HuR is a common mechanism employed by other tumors to promote tumor invasion and metastasis.

IL-8 is a target of oncogenic KRAS which initiates 90% of PDAC [29]. This cytokine is able to initiate multiple signaling pathways by activating two cell surface G-protein coupled receptors, CXCR1 and CXCR2, resulting in angiogenesis, tumor metastasis and tumorigenesis. Clinical studies have shown the up-regulation of IL-8 in pancreatic cancers is associated with an enhanced metastatic potential and overall poor prognosis [30, 32, 39–41]. In line with our *in vitro* findings displaying the effects of CRABP-II deletion on PDAC migration and invasion, IL-8 treatment did not affect PDAC cell growth but increased MMP-2 activity and enhanced cell invasion [31]. Besides MMP-2, MMP-9 [28] and MMP-14 [42] are also modulated by IL-8. Targeting IL-8 or its receptors CXCR1/CXCR2 has been shown to suppress tumor growth and induce cancer cell apoptosis in colon and breast cancer mouse models [43, 44]. However, inhibiting IL-8 directly may interfere with immune system and have serious side effects and safety risks [43]. Here, our findings of IL-8 regulation by CRABP-II provide a rationale of a potential novel treatment option to target CRABP-II rather than IL-8 and its receptor in PDAC and other cancers where IL-8 is positively regulated by CRABP-II.

Although IL-8/MMP-2/MMP-14 axis is shown as a downstream pathway of CRABP-II in this study, the underlying mechanism of CRABP-II up-regulation in PDAC remains unknown. In Wilms tumor and neuroblastoma, MycN binds to the E-box II at CRABP-II promoter and promotes its expression [11, 23]. In breast cancer, both AP-2 and RARα are able to drive CRABP-II expression through binding to the −5kb site of the promoter [45, 46]. In addition, epigenetic modification regulates CRABP-II expression [18, 23]. Further studies are warranted to examine the regulatory effect of Myc on CRABP-II expression in PDAC cells considering that Myc is a common downstream effector of KRAS and PI3K/AKT and plays a critical role in PDAC tumorigenesis and progression [47, 48]. Moreover, the evaluation of methylation status of CRABP-II promoter region in PDAC tumors and normal pancreatic ductal epithelial cells might also provide novel insight into the mechanism underlying CRABP-II overexpression in PDAC.

In summary, we identified a novel metastatic pathway in PDAC progression. Through cooperation with HuR, CRABP-II stabilizes IL-8 expression, which then enhances MMP-2/MMP-14 synthesis and promotes PDAC migration and invasion. Since CRABP-II over-expression is an early event in PDAC development, this molecular pathway provides new insight to a better understanding of PDAC metastatic behaviors and may also provide a novel interventional option.

## MATERIALS AND METHODS

### Cells, plasmids and antibodies

All lines including human pancreatic cancer cell lines, BxPC-3, Capan-1, Panc-1, Panc10.05 and 293T cells were obtained from ATCC. Panc-1 cells and 293T cells were maintained in Dulbecco’s modified Eagle’s medium (DMEM) supplemented with 4.5 g/liter glucose, 4.5 g/liter L-glutamine, 10% fetal bovine serum (FBS, Gibco) and 100 IU/ml penicillin/streptomycin. BxPC-3 cells and Panc10.05 cells were cultured in RPMI 1640 medium supplemented with 10% FBS or 15% FBS and 4 μg/ml human recombinant insulin (Invitrogen). All transfections were performed by using lipofectamine 3000 (Invitrogen). Charcoal stripped FBS with low hormone and retinoids was obtained from Gibco.

The pLKO.1 lentiviral vector harboring human CRABP2 shRNA (TRCN0000021373) and control luciferase shRNA (SHC007) were obtained from Sigma. The lentiviral CRISPR/Cas9 construct was generated by inserting CRABP2 guide sequence (AATCTCTGTGGTGCGCACGG) into BsmB I site of lentiCRISPRv2 vector (Addgene) and the CRISPR negative control plasmid was purchased from Santa Cruz. CRABP-II ORF was cloned to pCMV-3Tag-1 vector and the generated construct was further site-mutated at PAM sequence from TGG to TCG and used for rescue experiment.

Anti-CRABP-II, anti-MMP-14 mouse mAbs and protein A/G agarose beads were from Millipore (Billerica, MA). Anti-MMP-2 rabbit antibody, anti-HuR mouse mAb (3A2) and anti-Actin antibody were purchased from Santa Cruz Biotechnology. Anti-Flag M2 mAb was from Sigma and anti-Flag agarose beads from Clontech.

### qRT-PCR

qRT-PCR was performed by using two distinct reagents: Taqman (Invitrogen) or SYBR green (Bio-Rad). The Taqman probes used include CRABP2 (Hs00275636_m1), RARB (Hs00977140_m1), MMP2 (Hs01548727_m1), MMP9 (Hs00234579), MMP14 (Hs01037003_g1), TIMP2 (Hs00234278_m1) and 18s rRNA (4333760F). The primers used in SYBR green Q-PCR for IL8 (Hs.PT.58.38869678.g) and ACTB (Hs.PT.56a.40703009.g) were purchased from Integrated DNA Technology.

### MTT, wound healing and matrigel invasion assay

For cell proliferation assay, live cells were counted after trypan blue staining and seeded into 96-well plates at 3000 cell/well in quintuplicates. At day 1, 2, 3 and day 4, 20 μl of Celltiter96 AQueous One Solution Reagent (Promega) was added into wells and incubated with cells at 37°C for 3 hrs. The absorbance at 490nm (OD490) was immediately recorded with a 96-well plate reader (Bio-Rad). Wound healing assays and Matrigel invasion assays were performed as previously described [49].

### Stable cell line establishment

Both CRABP-II targeting shRNA and luciferase shRNA were packaged as lentiviruses in 293T cells according to standard protocol. Panc-1, BxPC-3 and Panc10.05 cells were infected and selected with 2.0, 0.5, 2.5 μg/ml of puromycin respectively for at least one week. The survival colonies in a whole plate were used as cell pools. For CRABP-II knockout, luciferase/GFP labeled Panc-1 cells were prepared as described previously [50]. Cells were then infected with lentiviruses harboring CRABP-II targeted CRISPR/Cas9 or non-targeting CRISPR negative control plasmids, and selected with 2.0 μg/ml of puromycin for two weeks. Stable colonies were identified by western blots. At least 5 knockout clones were combined and used as CRABP-II knockout (KO) cells. For rescue experiments, the pCMV-3Tag-CRABP-II construct with a nonsense mutation (TGG to TCG in PAM sequence) was transfected into CRABP-II knockout cells and selected with 1500 μg/ml of G418 for two weeks. Empty vector transfected cells were used as control.

### Gene expression microarray and data analysis

CRABP-II knockout cells and CRISPR negative control cells were cultured in hormone depleted media for 48 hrs before lysed for RNA isolation by using RNeasy Kit (Qiagen). RNA samples were reverse-transcribed, amplified and labeled using Illumina TotalPrep RNA Amplification Kit (Ambicon) according to the manufacturer’s instruction. cRNAs were hybridized to Illumina HumanHT-12 version 4 BeadChips. The expression profiles were corrected against background, quantile normalized and analyzed using a statistical R package, Limma [51]. The up- and down-regulated genes were identified by cutoff point at FDR adjusted *p*-value < 0.05 and fold change > 1.20. Pathway and function analysis based on those identified genes were performed using QIAGEN’s Ingenuity Pathway Analysis (IPA®, QIAGEN).

### Immunoprecipitation (IP) and RNA-immunoprecipitation (RIP)

Confluent Panc-1 cells were washed with ice-cold PBS and lysed in EBC buffer (50 mM Tris, pH8.0, 120 mM NaCl and 0.1% Triton X-100) supplemented with cocktail proteinase inhibitor and phosphatase inhibitor (Roche). Protein lysates were pre-treated with 100 μg/ml RNase (Qiagen) for 30 min, then mixed with mouse IgG isotype control or anti-HuR mouse mAb (3A2) and incubated at 4°C overnight. Mixture was then incubated with Protein G agarose beads for 1 hr, and washed and boiled in 2xSDS loading buffer. Precipitated proteins were separated and blotted with anti-CRABP-II antibody.

RNA immunoprecipitation (RIP) was conducted as described [52] with modification. In brief, flag-CRABP-II expressing cells and empty vector transfected cells were grown in hormone depleted media for 48 hrs and washed with ice-cold PBS. Cells were collected and lysed in polysome lysis buffer (10 mM HEPES, pH7.0, 100 mM KCl, 5 mM MgCl_2_, 25 mM EDTA, 0.5% IGEPAL) containing 2 mM DTT, RNase OUT (Invitrogen), proteinase inhibitors and 0.2 mg/ml Heparin. Cell extract was diluted in 10x volumes of NT2 buffer (50 mM Tris, pH7.5, 150 mM NaCl, 1 mM MgCl_2_ and 0.05% IGEPAL) and incubated with anti-flag beads at 4°C for 4 hrs. After 3-4 washes with ice-cold NT2 buffer, the beads and 100 ul of original cell extract (used as input) were mixed with Trizol reagents and isolated RNA was used for qRT-PCR.

### Pancreas orthotopic transplantation and bioluminescence imaging

Nude mice (6-8 weeks) were obtained from Case Medical School Animal Facility and the animal research procedure described here was approved by the Institutional Animal Care and Use Committee. Mice were anesthetized with a single intraperitoneal injection of 1.25% avertin. Pancreas was exposed through a left abdominal incision. Sub-confluent CRABP-II knockout cells and control cells were detached by trypsin-EDTA and cell viability was assessed by trypan blue staining. Two million cells were resuspended in 50 μl of 1:1 mix of media and ice-cold Matrigel and injected into pancreas. After transplantation, pancreas was carefully returned to the peritoneal cavity and the opening was sutured. Tumor growth was monitored every two weeks by bioluminescence imaging with IVIS spectrum *in vivo* imaging system (PerkinElmer).

## SUPPLEMENTARY FIGURES


