# Supplementary material for: CRABP-II enhances pancreatic cancer cell migration and invasion by stabilizing interleukin 8 expression
Source: Oncotarget. 2016 Dec 26;8(32):52432–44. doi: 10.18632/oncotarget.14194 (PMC5581040; doi:10.18632/oncotarget.14194)
Supplement: Supplementary file 1 [file oncotarget-08-52432-s001.pdf]

CRABP-II enhances pancreatic cancer cell migration and invasion by stabilizing interleukin 8 expression

SUPPLEMENTARY FIGURES

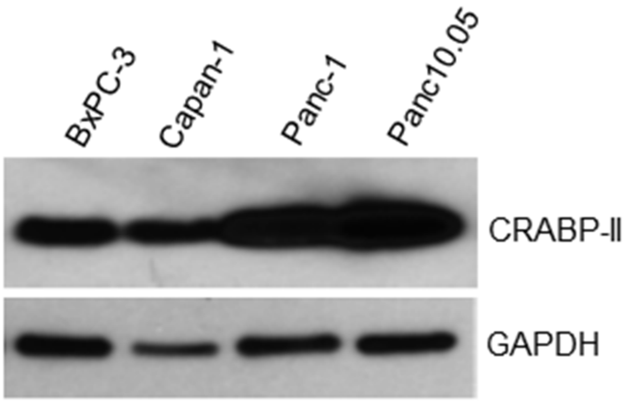

Supplementary Figure 1: Expression of CRABP-II in PDAC cell lines. Proteins in whole cell lysis of denoted PDAC cell lines were immunoblotted by anti-CRABP-II antibody and GAPDH was used as loading control.

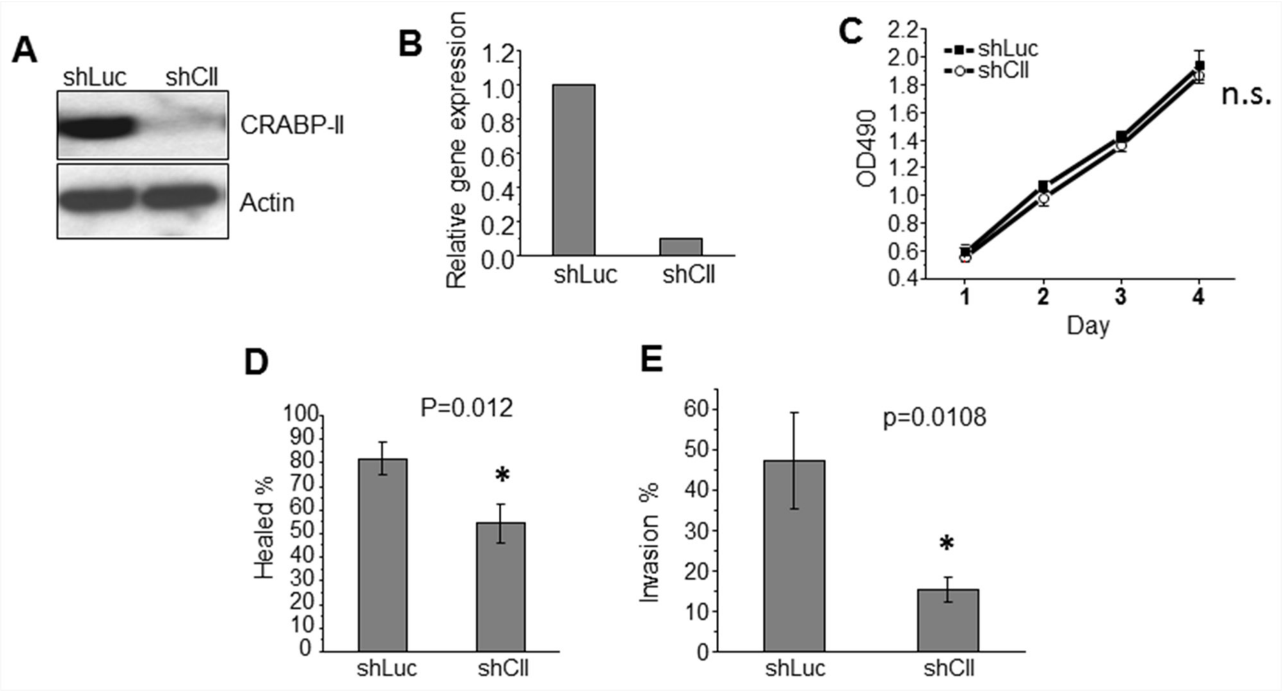

Supplementary Figure 2: Knockdown CRABP-II does not affect cell proliferation, but reduces cell migration and invasion. A. and **B.** Western blot and qRT-PCR showing down-regulation of CRABP-II by shRNA. Luciferase shRNA was used as a control. **C.** MTT assays showing no difference of cell proliferation between CRABP-II KD cells and control cells. n.s. means no significant difference by one-way AVONA test. **D.** Wound healing assays showing the decrease of cell migration in KD cells. **E.** Matrigel invasion assays showing the reduction of cell invasion in KD cells. Data shown in D and E represent the means ± SD of three independent experiments. * p< 0.05 by student t-test.

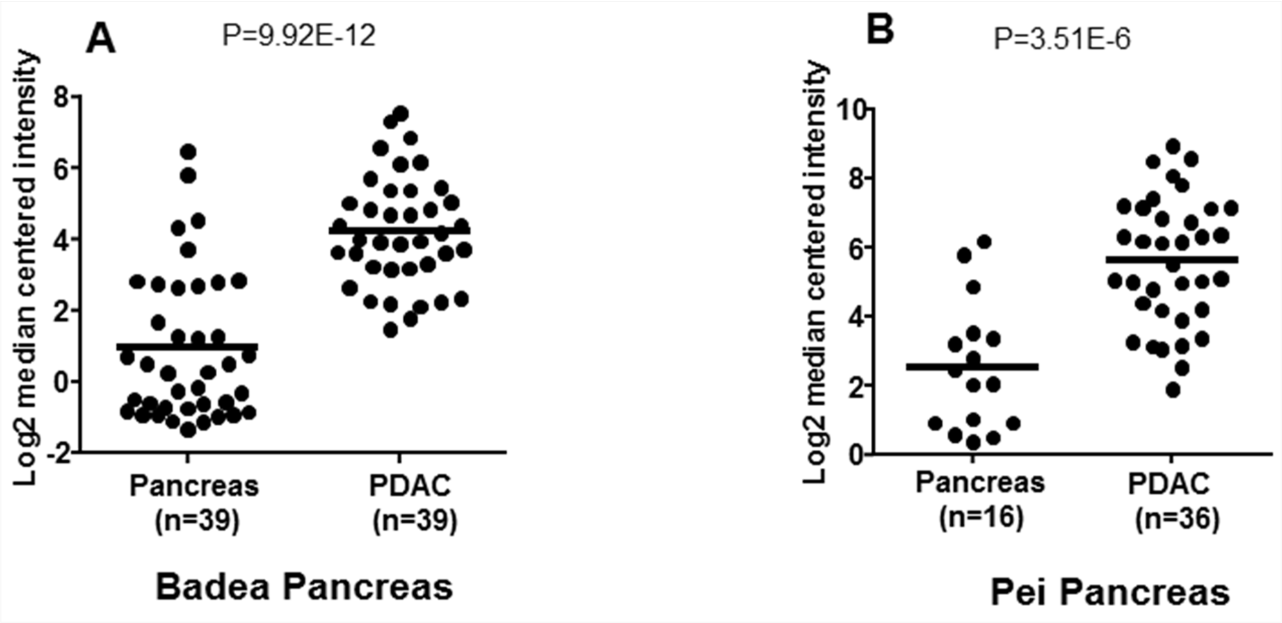

Supplementary Figure 3: Overexpression of IL-8 in pancreatic cancer. A. IL-8 expression in Badea Pancreas microarray database from Oncomine; ** p = 9.92E-12 by student t-test. **B.** IL-8 expression in Pei Pancreas microarray database from Oncomine; ** p = 3.51E-6 by student t-test.

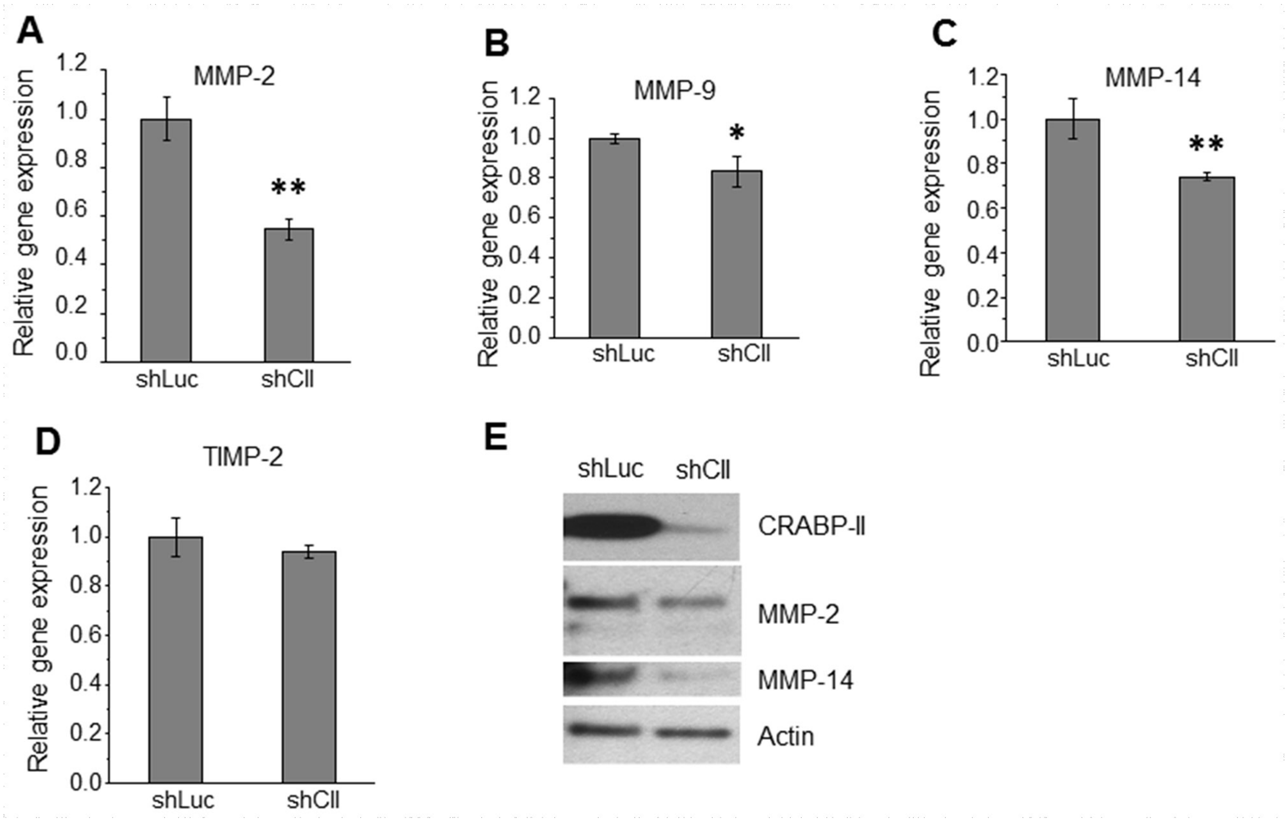

Supplementary Figure 4: Down-regulation of MMP2, MMP14 expression by CRABP-II silencing. Data were assessed by qRT-PCR and western blots. Data shown in **A, B, C.** and **D.** represent the means ± SD of three biological replicates. ** p< 0.01, * p<0.05 by student t-test.

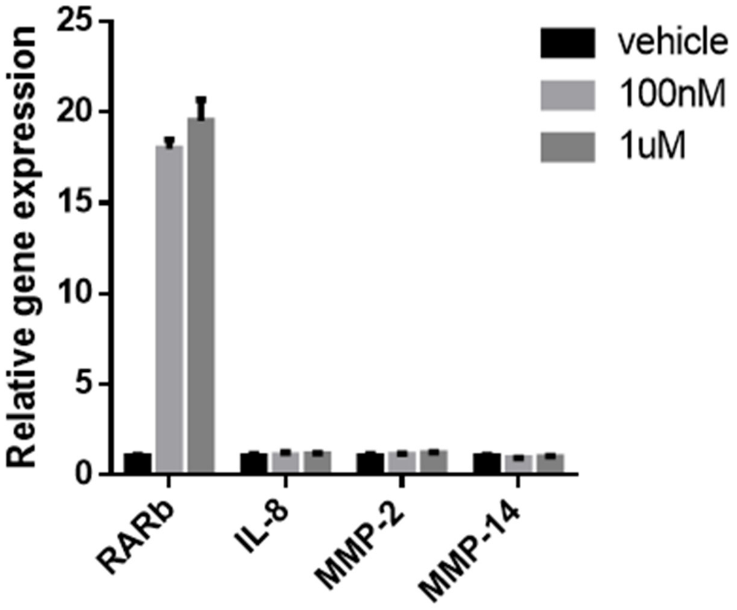

Supplementary Figure 5: Retinoic acid (RA) independence of IL-8, MMP-2 and MMP-14 expression in Panc-1 cells. Cells were treated with denoted doses of RA (0, 100, 1000 nM) for 8 hrs. The expressions of IL-8, MMP-2 and MMP-14 were assessed by qRT-PCR. RAR-β was used as a positive control. Data represent the means ± SD of triplicate wells from three independent experiments.

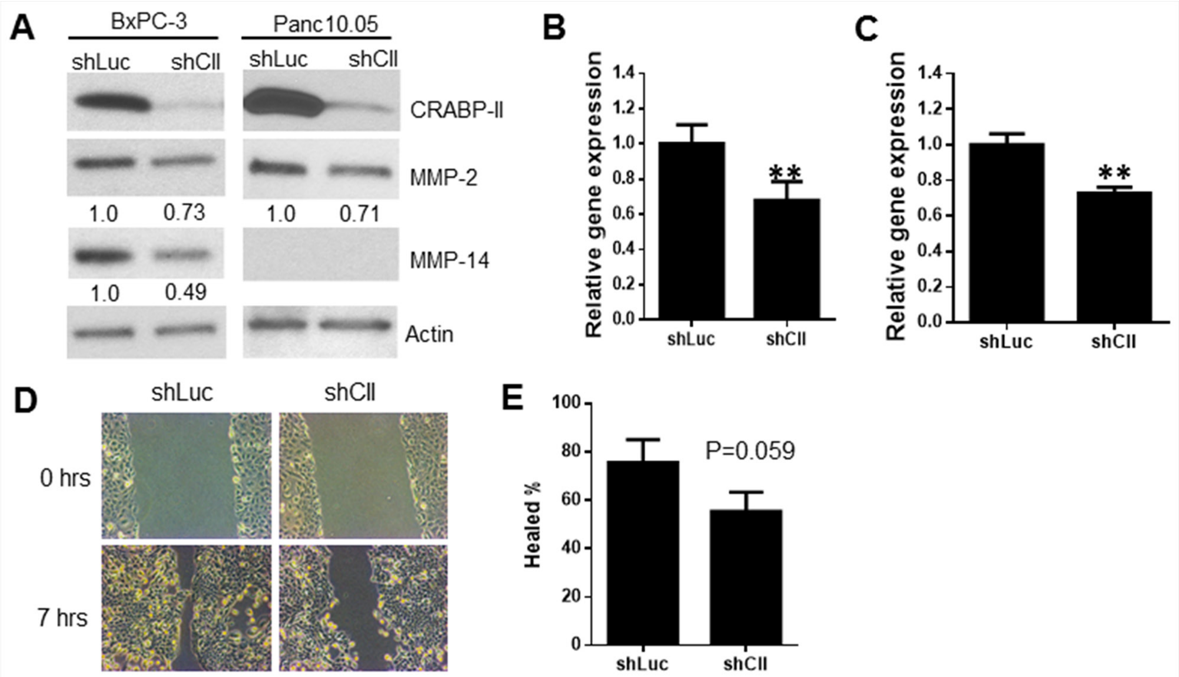

Supplementary Figure 6: Knockdown CRABP-II in BxPC-3 and Panc10.05 cells. A. Western blot showing down-regulation of CRABP-II by shRNA in BxPC-3 and Panc10.05 cells. Luciferase shRNA was used as a control. MMP-2 and MMP-14 expression were shown as denoted. **B.** and **C.** qRT-PCR showing the decrease of IL-8 expression by CRABP-II silencing in both BxPC-3 (B) and Panc10.05 (C) cells. Data shown represent the means ± SD of three biological replicates. ** $p < 0.01$ by student t-test. **D.** and **E.** Wound healing assays showing the decrease of cell migration in CRABP-II KD BxPC-3 cells. Data shown represent the means ± SD of three independent experiments. $p=0.059$ by student t-test.

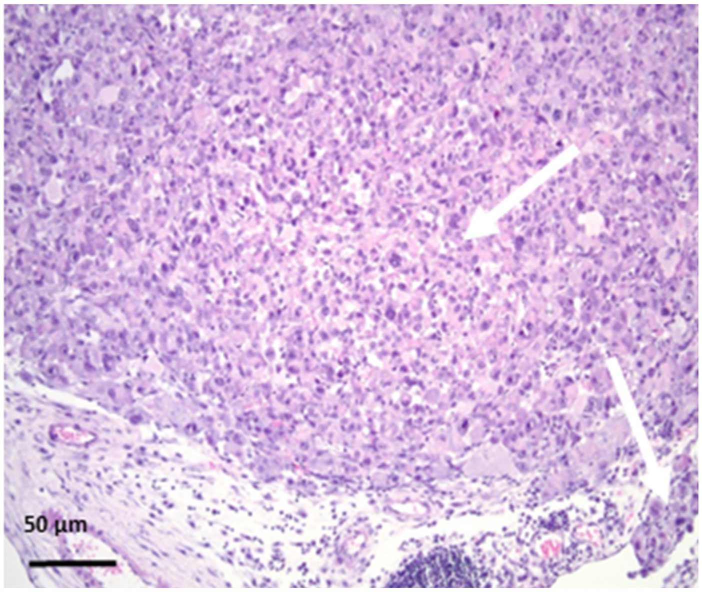

Supplementary Figure 7: Lymph node metastasis in orthotopic mouse model. As described in Materials and Methods, the luciferase expressing CRABP-II knockout cells and CRISPR negative control cells were orthotopically injected into pancreas of nude mice. Mice were scarified at 11 weeks after injection, and tumors and metastatic lymph nodes were separated, fixed and H&E stained. Picture showing tumors in lymph node from WT cell injected mice. Arrows denote the metastatic tumors. None metastatic lymph node was found in CRABP-II KO cell injected mice.
